# Proton-Coupled Electron-Transfer Mechanism for the Radical Scavenging Activity of Cardiovascular Drug Dipyridamole

**DOI:** 10.1371/journal.pone.0039660

**Published:** 2012-06-22

**Authors:** Abolfazl Barzegar

**Affiliations:** 1 Research Institute for Fundamental Sciences (RIFS), University of Tabriz, Tabriz, Iran; 2 The School of Advanced Biomedical Sciences (SABS), Tabriz University of Medical Sciences, Tabriz, Iran; University of Connecticut, United States of America

## Abstract

Dipyridamole (DIP) is a well-known pharmaceutical drug used as a coronary vasodilator and anti-platelet agent in clinics for treating several cardiovascular diseases. Primarily, the therapeutic effects of the drug are attributed to its antioxidant potency. In this research, we aim to declare the unknown antioxidant mechanism of DIP as well as its potent chain-breaking antioxidant activity in polar aqueous medium inside the cells, using different experimental methods and theoretical quantum calculations. Data demonstrated the higher antioxidant capacity of DIP against ROS and free radicals in polar cell's interior. DIP is capable of generating long living and noninvasive DIP^•^ radicals in oxidant condition that leads to an effective “chain-breaking antioxidant” activity. Quantum computational data indicated that DIP antioxidant has more favorable ionization potential than trolox which means DIP has higher antioxidant activity. Also, data showed that the direct hydrogen-transfer is not a favorable process to construct DIP^•^ because of high barrier energy, though electron-transfer process can more easily to produce DIP^•+^ with the lowest barrier energy. Altogether, the electron donating potency of DIP to reduce ferric ion, having the low anodic oxidation peak potential, producing long lived stable DIP^•^ radicals and protecting myoblast cells from oxidation, proposed the excellent “chain-breaking antioxidant” potency via electron-transfer mechanism of this vasodilator DIP drug in polar aqueous medium.

## Introduction

**Figure 1:**
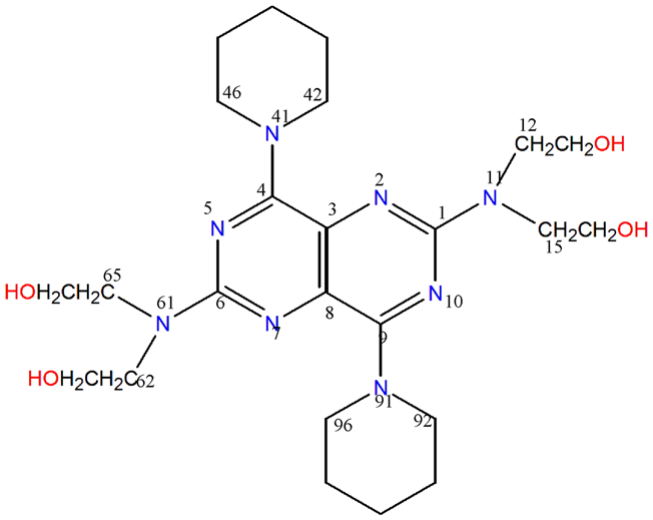
Chemical structure of dipyridamole (DIP).

Dipyridamole (DIP) [2,6-bis(diethanolamino)-4,8-di-piperidinopyrimido-(5,4-d)pyrimidine] is a dilatation agent of coronary artery used as a treatment for several cardiovascular diseases such as angina pectoris and myocardial infarction [1], [2]. The drug can be easily adsorbed in the human body and entered into the blood. It prevents blood cells called platelets from clumping together inside the blood vessels and reversibly, inhibits platelet aggregation and platelet-mediated thrombotic disease [3]. Platelet and vascular stimulation leads to release of reactive oxygen species (ROS) that are known for influencing vascular reactivity and thrombosis [4]. It has been hypothesized that the therapeutic effects of DIP are attributed to its antioxidant potency because of having special chemical structure [3]. The structure is shown in Figure 1.

DIP has antioxidant properties which could stabilize platelet and vascular membranes as well as prevent the oxidation of LDL molecules [5]. Studies have demonstrated that the drug is an efficient chain-breaking antioxidant that inhibits lipid peroxidation [6], scavenges superoxide and hydroxyl radicals [7], [8] protects erythrocyte Times New Roman s membranes from oxidation [9] and promotes angiogenesis in diabetic mouse hind limb model [10]. The affinity of DIP for lipid phase is related to the fact that it acts as an inhibitor for membrane peroxidation [11] and also lipid peroxidation by scavengeing hydroxyl HO^•^ and lipid peroxyl ROO^•^ radicals in lipid phase [12], [13]. Using different lipid system models, such as micelles or monolayers including DIP, it was indicated that DIP is abundant in lipid systems [14]. It appeared interesting to study the direct influence of DIP on ROS protection as to the activity is known to be dependent on membrane surroundings [14], [15]. These findings obviously indicated that the strong antioxidant affectivity of DIP in protecting lipids from peroxidation is associated with the higher hydrophobicity of the compound [16]. Although this drug has been used for many years, its mechanism of action is still unclear. However, the potent chain-breaking antioxidant activity of DIP in polar aqueous medium especially intracellular condition has not been reported so far. In order to better understanding, the antioxidant potency of DIP has been extensively studied using electrochemical oxidation reactions in phosphate buffer, optical behavior, ferric ion reduction, potent to produce the stable radical, intracellular (L6-myoblasts) ROS protection against toxic hydroperoxide compounds as well as quantum chemical computations. Data declared the higher antioxidant capacity of DIP in polar intracellular to reduce ROS compounds through electron transfer mechanism.

**Figure 2:**
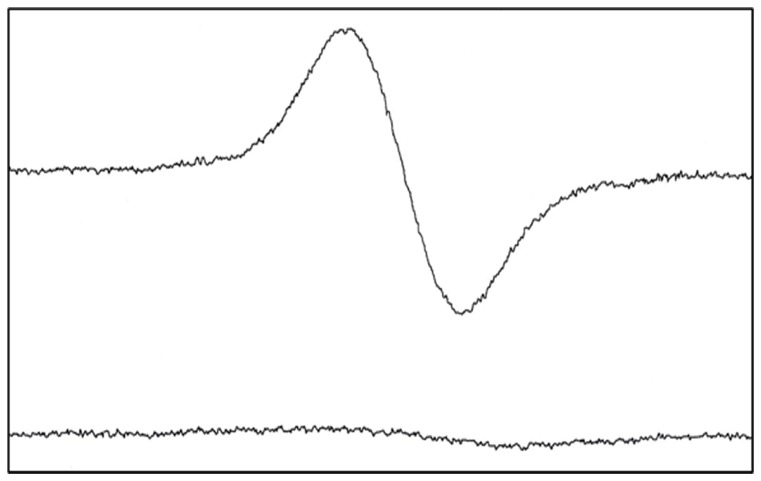
Room temperature EPR spectrum of the DIP^•^ radical in DMSO. DIP^•^ radicals were produced by the reaction of 10 mM DIP with 500 mM of cumene hydroperoxide in the presence of FeSO_4_ (fenton reaction). Bottom spectrum indicated all reagents without having DIP. It should be noted that because of technical problem it was impossible to detect DIP^•^ radicals in PBS. Detecting DIP^•^ radicals needed to use high concentration of DIP (10 mM) whereas DIP was completely insoluble in PBS at this concentration.

**Figure 3:**
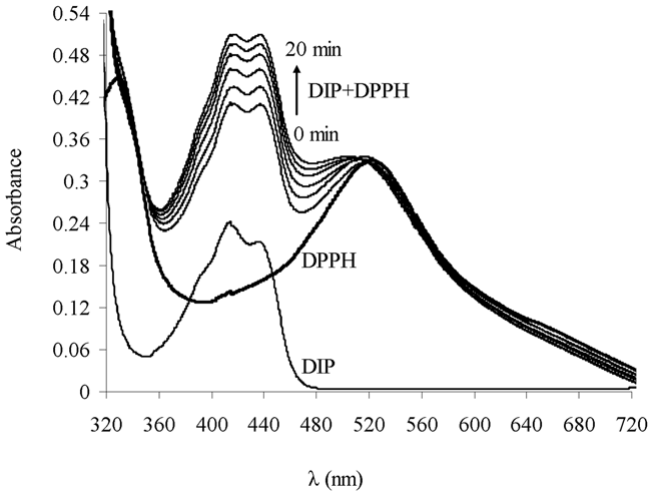
Optical absorption spectra of DIP in DMSO. The mentioned DIP and DPPH^•^ spectra indicate 30 µM of each compound alone in DMSO. During 20 min, the behavior of DIP spectra followed by adding DPPH^•^ radical (mentioned DIP+DPPH^•^).

## Materials and Methods

The reagents used in this work were of analytical grade that obtained from Sigma Aldrich (diprydamol, cumene hydroperoxide, trichloroacetic acid and 2,2-Diphenyl-1-picrylhydrazyl radical) and Merck (potassium ferricyanide, ferric chloride, disodiumhydrogen phosphate dehydrate, phosphoric acid). 2′,7′*-*dichlorodihydrofluorescein diacetate (DCFH_2_-DA) was provided by Molecular Probes (Eugene, OR). Fetal bovine serum was from GIBCO (Grand Island, NY). Dulbecco's modified Eagle's medium (DMEM), antibiotics and sterile plasticware for cell culture were from Flow Laboratory (Irvine, UK). Water treated in a MicroMed-TKA system (conductivity <0.1 µS cm^−1^), was used to prepare the solutions. All chemicals were used without previous purification. Stock solution of DIP was prepared by dissolving it in DMSO at a concentration of 10 mM. The solution was stored at 4°C in dark to avoid any decomposition.

### EPR measurements

The radical DIP^•^ was generated by oxidation of DIP in fenton reaction. The solution was carefully deoxygenated by bubbling nitrogen. The solutions were drawn into glass capillaries, sealed and then measured using an ESP300 instrument (Bruker Spectrospin, Karlsruhe, Germany) equipped with a high sensitivity TM110 X-band cavity.

**Figure 4:**
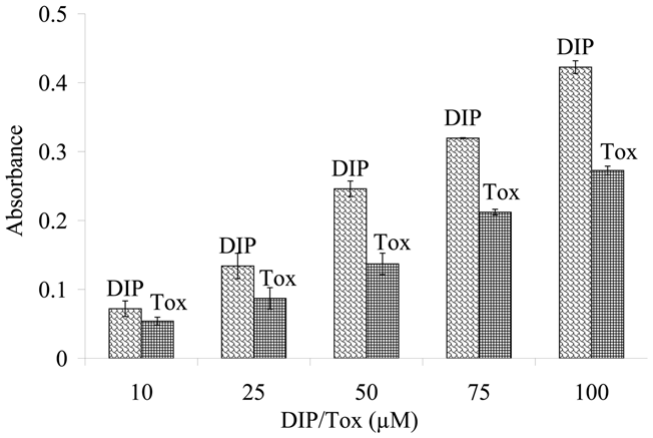
Electron donating capacity of DIP and trolox (Tox) to ferric iron (Fe^3+^) reduction. The absorbance at 700 nm was recorded as a function of DIP and Tox concentrations in phosphate buffered saline (PBS) to monitor the reduction of ferric ion to ferrousion.

**Figure 5:**
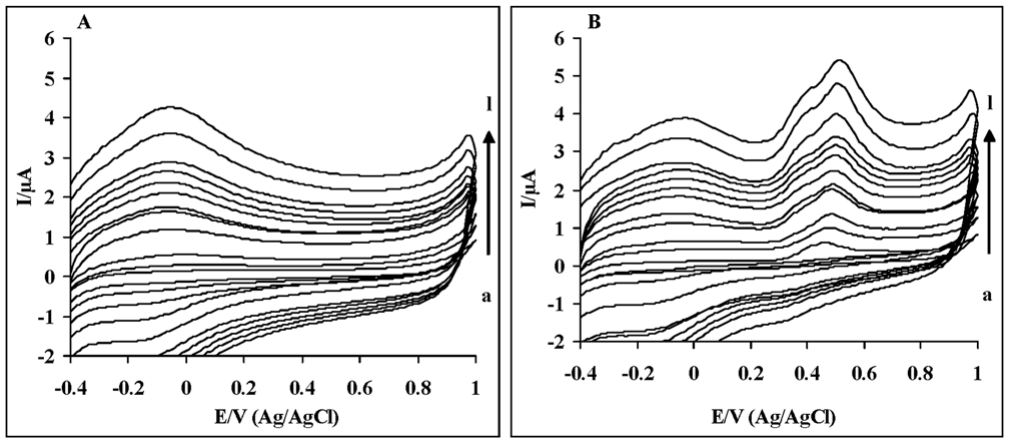
Cyclic voltammograms of DIP oxidation at the surface of glassy carbon electrode for different scan rates (a–l): 100, 200, 300, 400, 500, 600, 700, 800, 900, 1000, 1250, 1500 mVs−1. Voltammograms depicted in 0.2 M phosphate buffer solution (pH 7.4) in the absence of DIP (panel A) and the presence of 0.1 mM DIP (panel B). In the absence of DIP, no apparent cyclic voltammetric signals are observed in the phosphonate buffer solution. DIP exhibits anodic peak at 0.5 V.

**Figure 6:**
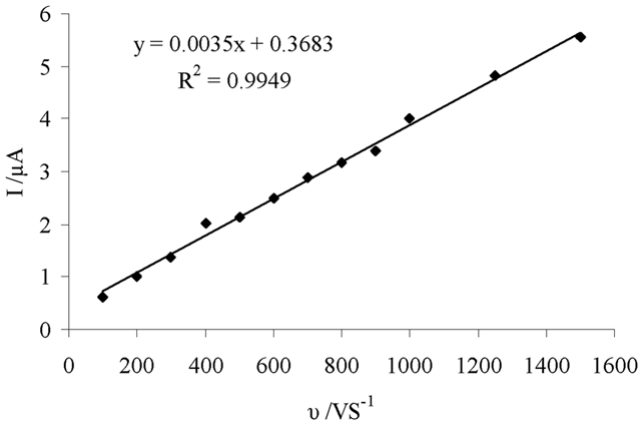
The plot of oxidation peak current (I) of DIP at 0.5 V vs. scan rates (υ). It shows linear relationship between the scan rate and the peak current of 0.1 mM DIP at the glassy carbon electrode.

### UV-Vis spectrophotometer method

0.5 mL of 30 µM solution of 2,2-Diphenyl-1-picrylhydrazyl radical (DPPH^•^) in DMSO was thoroughly mixed with 0.5 mL of 30 µM of DIP. DPPH is widely used to evaluate the ability of compounds to act as free radical scavengers [17], [18], [19]. The optical behavior of DIP in the presence of DPPH^•^ was studied for different incubated times.

### Ferric ion reduction

Iron in the +3 oxidation state (Fe^3+^) is known as the ferric ion. The process by which the ferric ion reduces to the +2 oxidation ferrous ion state (Fe^2+^) is known reducing power assessment. The reducing power of DIP was determined by analyzing its electron donor potency according to the method we have more recently explained [20]. Potassium ferricyanide (1%) was shortly incubated with different concentrations of DIP for 30 min at 50°C in phosphate buffered saline (PBS). After adding trichloroacetic acid (10%) and FeCl3 (0.1%) the absorbance at 700 nm was recorded as reducing power of DIP in PBS solution.

### Cyclic voltammetry experiments

The electrochemical measurements were carried out using a potentiostat-DropSens model μStat 400. A conventional three-electrode cell containing a glassy carbon working electrode, platinum wire counter electrode and Ag/AgCl (3.0 M KCl) reference electrode were employed for the voltammetric application. Electrochemical experiments were carried out in a conventional electrochemical cell, containing 2 mL of phosphate buffer solution and 0.1 mM of DIP. Phosphate buffered solution (pH 7.4, 0.2 M) was prepared by adding phosphoric acid in 0.2 M Na_2_HPO4 solution. Voltammograms were recorded with the potential sweeping from –0.4 to 1.0 V at different scan rates.

**Figure 7:**
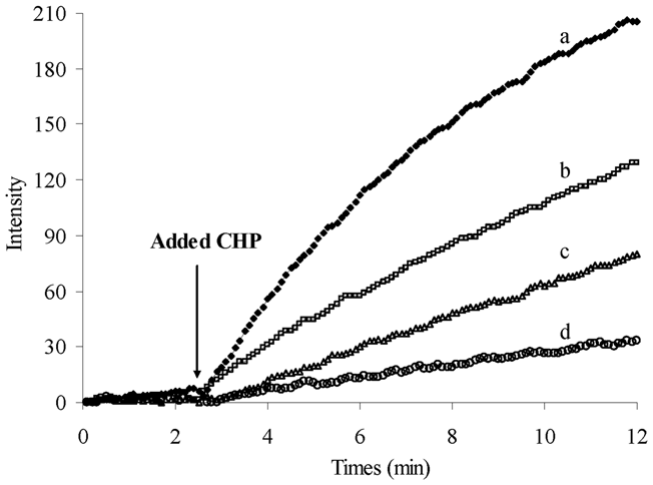
Intracellular ROS determination by DCF method. Samples were first incubated (30 min) with different concentrations of DIP; a:0.0, b:0.25, c:0.5, d:1.0 µM. The changes of fluorescence intensity were monitored for the following intracellular ROS. The fluorescence behavior of the samples was monitored by excitation at 498 nm and emission 530 nm. Samples were induced to produce intracellular ROS and radical generator by adding the ROS stimulating agent of cumene hydroperoxide (CHP) that was mentioned by arrow. The final concentration of CHP was 300 μM by adding 10 μL of CHP in the final volume of 1 mL.

**Figure 8:**
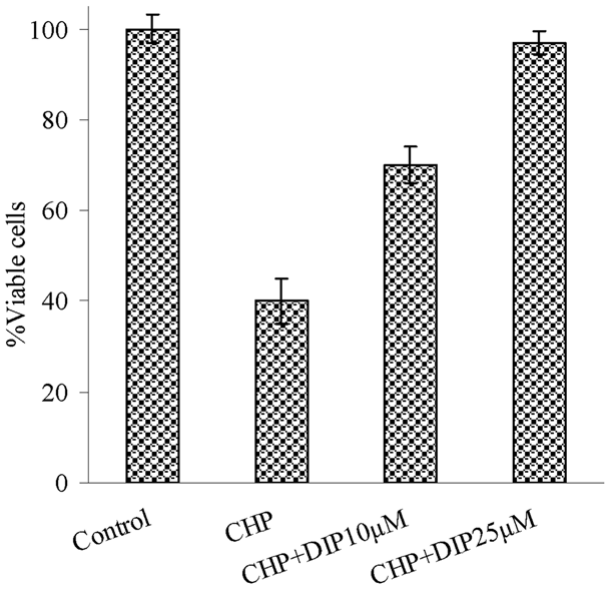
Cell viability against cytotoxic effect of CHP using MTT assays. L-6 myoblasts exposed to cumene hydroperoxide (CHP) after 30 min treatment of cells with different concentration of DIP (0, 10 and 25 µM). Control refers without any addition of DIP and CHP. No detectable toxic effect on cell viability has been recorded for DIP in different concentrations.

### Cell cultures

The skeletal rat muscle L-6 myoblast cells were obtained from the American Type Culture Collection (Rockville, MD). Cells were seeded in 75-cm^2^ flasks for tissue culture and grown in DMEM supplemented with 10% fetal bovine serum, 100 mg mL^−1^ streptomycin and 100 U mL^−1^ penicillin, in an atmosphere of 5% CO_2_ at 37°C.

### Intracellular ROS assay

Principle of the intracellular ROS evaluation was exploited via DCF method that we have published more lately [21]. Briefly, the cells were incubated with 10 µM DCFH_2_-DA in phosphate buffered saline (PBS) at 37°C for 30 min then washed twice with PBS (37°C ). The oxidized DCF under intracellular ROS caused cells staining to be easily detectable by fluorimeter.

### Cell viability assay

Cell viability assays were done in order to evaluate the antitoxic capacity of DIP against cytotoxic cumene hydroperoxide (CHP) compound. Myoblast cell samples were treated with different concentrations of DIP for 30 min. Then cells were induced by CHP as a powerful cytotoxic compound for 30 min. The percentage of viable cells in the presence and absence of DIP was determined by the methylthiazole tetrazolium (MTT) assay [21], [22].

**Table 1:** Ionization potentials (IPs) of DIP and trolox (Tox).

Compound	IP (kcal/mol)	ΔIP^a^ (kcal/mol)
**DIP**_(**gas phase**)_	132.00	−51.99
**DIP**_(**water effect**)_	131.77	−52.22
**Tox**	152.10	−31.99

Parameters have been computed using B3LYP/6-31G(d)//AM1 method without and with solvent (water) effect. The molecular geometries were first optimized in gas phase then the solvent effect has been estimated in the frame of Onsager model to calculate the single-point energy. ^a^Relative to 184.09 kcal/ mol for phenol in the same basis set.

**Figure 9:**
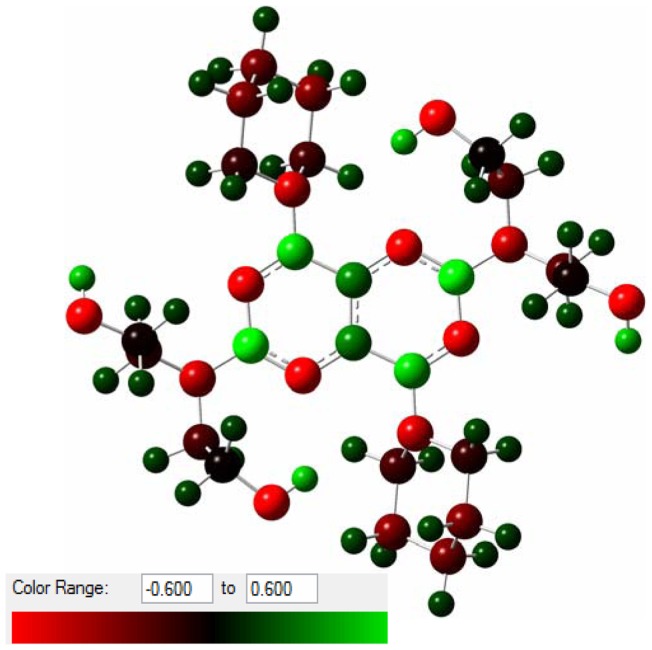
Optimized structure of dipyridamole. Atomic charges indicated by colors in the range of −0.6 (red) to +0.6 (green).

**Table 2:** Some chosen geometrical parameters including H bonding, bond angles and relevant torsion angles for 3D structure of DIP. Calculated parameter values using AM1 were compared with computation PM3//B3LYP/6-31G* values by Borges et al. [35], as well as crystal X-ray [36].

Parameters	AM1	PM3//B3LYP/6-31G* [35]	Crystal Structure [36]
**Hydrogen bonding** (**A°**)			
**O**(**14**)**–H**(**14**)**···N**(**2**)	2.569	2.590,1.923	2.778, 2.829
**Bond angles** (**Deg.**)			
**N**(**2**)**–C**(**1**)**–N**(**11**)	118.5	118.3,118.0	119.2, 117.7
**N**(**10**)**–C**(**1**)**–N**(**11**)	117.1	-	114.2, 116.4
**C**(**1**)**–N**(**2**)**–C**(**3**)	117.3	-	116.5,115.5
**N**(**2**)**–C**(**3**)**–C**(**4**)	121.3	121.1,120.9	121.4, 120.7
**N**(**2**)**–C**(**3**)**–C**(**8**)	123.19	-	121.3, 123.6
**C**(**4**)**–C**(**3**)**–C**(**8**)	115.4	-	116.3, 115.5
**C**(**3**)**–C**(**4**)**–N**(**5**)	120.7	-	119.9, 120.5
**C**(**3**)**–C**(**4**)**–N**(**41**)	118.40	121.7, 21.6	122.2, 122.1
**N**(**7**)**–C**(**6**)**–N**(**61**)	117.10	118.6, 116.3	115.5,114.5
**C**(**62**)**–N**(**61**)**–C**(**65**)	116.0	116.6, 120.6	120.4, 120.8
**C**(**12**)**–N**(**11**)**–C**(**15**)	116.0	116.4,119.2	119.8, 120.2
**C**(**42**)**–N**(**41**)**–C**(**46**)	112.7	112.7, 112.9	112.3, 113.1
**C**(**92**)**–N**(**91**)**–C**(**96**)	112.7	112.7,112.9	113.6, 112.7
**Dihedral angle** (**Deg.**)			
**N**(**2**)**–C**(**1**)**–N**(**11**)**–C**(**12**)	11.0	10.3, −2.6	−7.3, 9.8
**N**(**10**)**–C**(**1**)**–N**(**11**)**–C**(**15**)	−25.8	−22.0, −1.7	−1.9, −0.7
**N**(**5**)**–C**(**6**)**–N**(**61**)**–C**(**65**)	−25.7	16.1, −4.0	1.3, 1.5
**N**(**7**)**–C**(**6**)**–N**(**61**)**–C**(**62**)	11	−14.5, −2.3	1.8, −1.6
**N**(**11**)**–C**(**12**)**–N**(**13**)**–C**(**14**)	−124.6	−117.5, −86.8	−73.6,74.4(−167.5)
**N**(**11**)**–C**(**15**)**–C16**)**–C**(**17**)	−177.5	−74.9, −63.6	−60.0, 69.7
**C**(**3**)**–C**(**4**)**–N**(**41**)**–C**(**42**)	73.5	65.6, 47.5	50.2, −51.2
**C**(**8**)**–C**(**9**)**–N**(**91**)**–C**(**96**)	73.5	66.5, 30.4	51.1, −52.4
**N**(**8**)**–C**(**9**)**–N**(**91**)**–C**(**92**)	−153.00	−156.7, −169.4	−159.9, 163.4

### Theoretical studies

Quantum chemical calculations were aimed to determine the molecular geometry and energetic properties associated with antioxidant parameters of the molecule. For this purpose, density-functional-theory (DFT) method [23] was used to calculate ionization potential (IP) which takes advantage of accuracy and economy [24]. Three dimensional structure of DIP was obtained from NCBI PubChem Compound database (CID: 3108) [25]. The molecular geometries were first optimized by the molecular-mechanic MM+ method and followed by the semiempirical quantum-chemical method AM1 [26]. Then, B3LYP functional on basis sets of (U)B3LYP/6-31G(d) and RO)B3LYP/6–31G(d) was used to calculate the single-point energy (SPE). UB3LYP and ROB3LYP refer to the unrestricted Open-Shell and restricted Open-Shell calculation that used for the radical generated after electron-abstraction (cation radicals) and H-abstraction respectively. The molecular energy consists of (U)B3LYP/6-31G(d)-calculated SPE and AM1-calculated zero-point vibration energy (ZPVE, scaled by a factor of 0.973) [24], [27]. The ionization potential was defined as IP  =  Ec – Ep, where Ec is the energy of the cation radical and Ep is the energy of the parent molecule. Also, in this work, we investigated polar solvents effects on the electronic structural properties of DIP within the Onsager self-consistent reaction field (SCRF) model using a DFT method [28], [29]. Using Onsager solvent model in water (e = 80) and the cavity radius were calculated by the Gaussian program. Onsager–SCRF method is efficient in taking account of long-range solute–solvent electrostatic interaction [30]. The molecular energy in water consists of (U, RO)B3LYP/6-31G(d)-calculated SPE energy. All of the quantum-chemical calculations were carried out using Gaussian 98 program [31].

**Figure 10:**
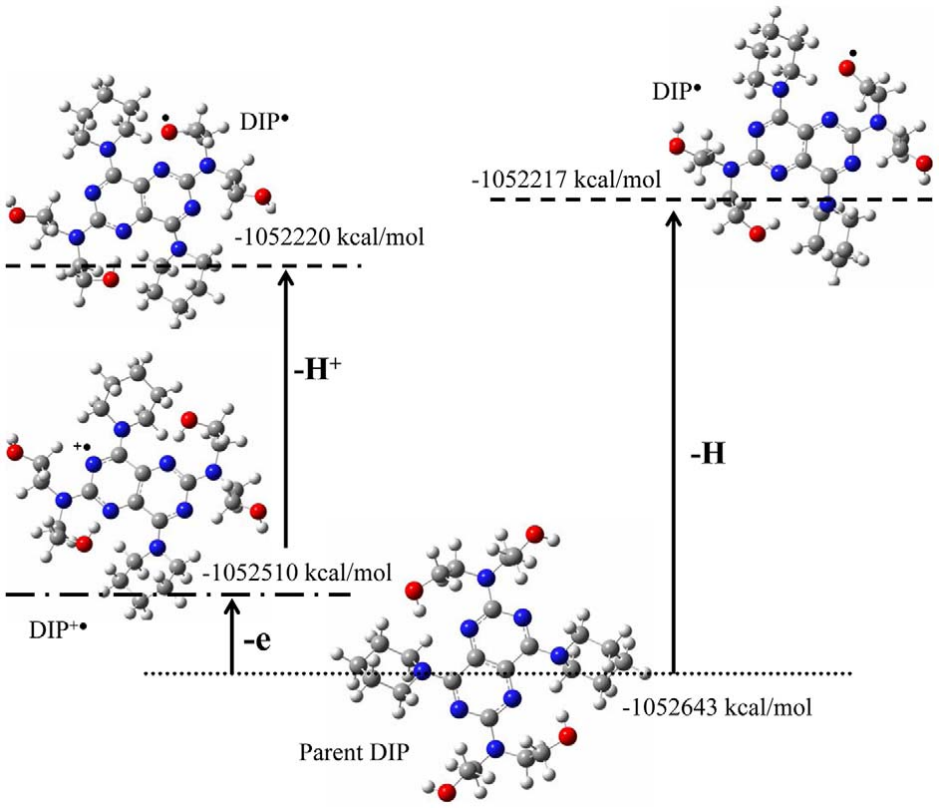
Structure and relative energies of DIP as well as its different derivative radicals. DIP^•+^ involves cation radical that derived from electron transfer, DIP^•^ is final radical involves proton donation (left geometry) and radical derived H atom abstraction via direct HAT reaction (right geometry).

## Results

### Production of stable DIP^•^ radical

DIP molecules were reacted with cumene hydroperoxide (CHP) in the presence of iron (II) ions which stimulate radical generation known as Fenton's reaction method [21]. The iron metal has a strong catalytic power to generate highly reactive hydroxyl radicals (^•^OH). After addition of the iron and CHP, they will react together generating some hydroxyl radicals as:

(1) Fe^2+^ + CHP → Fe^3+^ + ^•^OH + CHO^−^ (Cumene hydroxide)

When mixing the reactants, the solution shows an EPR spectrum (Figure 2) denoting generation of DIP^•^ radical. Free radicals are generally very reactive and unstable structures because of having an unpaired electron that temporarily disappear by reacting with each other or substrates in the presence of liquids. Accordingly, it is really hard to trace free radicals in solution. Hence, the only way to detect the intermediate DIP^•^ radical in the oxidant condition is practically possible for the long living stable DIP^•^ radical. The production relatively stable free radical, DIP^•^, denotes that DIP^•^ has no tendency to react with other substrates in solution. This excellent property should give effective chain-breaking antioxidant activity for DIP in biological environment which is expected to has the least side effects from DIP^•^ radical reactions (see discussion).

### Electronic absorption spectra

The structure of DIP, shown in Figure 1, presents a stable hetero-aromatic double ring core, which is responsible for the characteristic UV-Vis absorption. The spectroscopic behavior of DIP through the absorbance studies confirmed the result of EPR. In the spectral range between 320–720 nm, two bands including 418 nm and 442 nm are observed for DIP in the absence of DPPH^•^ radical (Figure 3). The change of bands' shape was concomitant with adding DPPH^•^ radicals. Data in Figure 3 indicated the intensity of second band (442 nm) which got increased to be equal with the first band (418 nm) in the presence of DPPH^•^. Moreover, during the incubation periods (0 to 20min), the intensity of the bands (418 and 422 nm) increased. These data indicated the creation of a new compound that we detected by EPR (Figure 2). Therefore, the change of bands' shape with increasing of the absorbance intensity of the bands during incubation of DIP with DPPH^•^ denote the production of stable DIP^•^ radicals.

### Electron donating effectiveness of DIP

Evaluation of electron donating basically gives clear insights into reducing potency of antioxidants against oxidative agents. This method is based on the ability of the DIP to reduce Fe^3+^ to Fe^2+^. Since reduced ferrous ion is green in color, increased reducing power of the sample causes the absorbance of the mixture to be increased at 700 nm. Figure 4 shows that there is good correlation between DIP concentrations and amounts of reduced Fe^3+^ in phosphate buffered solution. Data obviously indicated a higher electron donating ability for DIP than trolox (the water-soluble vitamin E analogue). Hence, DIP has an electron donating capacity which is active in polar phosphate buffer medium. These findings propose that DIP should be active in polar intracellular location in order to suppress high active ROS compounds (see next sections).

### Electrochemical oxidation of DIP

In order to confirm the reliability of the previously described assessment of electron donating potency of DIP, in this part, the electrochemical oxidation of DIP in phosphate buffer has been studied using cyclic voltammetry. Cyclic voltammetry is a widely used electroanalytic technique that allows the redox properties of molecules such as antioxidants in a solution to be determined [32]. Figure 5 is a repetitive cyclic voltamperogram for DIP, recorded for the large range of scan rates. No apparent cyclic voltammetric signals were observed in the phosphonate buffer solution in the absence of DIP for the different scan rates. As shown in Figure 5, the DIP compound exhibits clearly anodic oxidation peak potential (∼0.5 V) in phosphate buffer (pH 7.4). Electro-oxidation technique can be used for evaluating the electron-donating capacity of the molecule. Therefore, anodic low oxidation peak potential of DIP indicates the easily electron donating potency of this compound.

Peak currents (I) vs. scan rates (υ) give useful information about both phenomena including: diffusion controller or adsorption controller of oxidation process of solutes by electrode. Figure 6 shows the plot of anodic peak current of linear sweep voltammetry vs. scan rates. The linearity of plot in the range of 100–1500mVs^−1^ indicates that the oxidation of DIP at the glassy carbon electrode is rather an adsorption-controlled than a diffusion controlled process.

### Intracellular ROS protection potency of DIP

L-6 myoblasts were used as an in vitro cell model to investigate the intracellular efficiency of DIP as a protective agent against ROS damage. These cells are good candidates and easy to use as an in vitro model for testing the ability of different types of antioxidants to scavenge intracellular ROS via DCF method [21], [33]. DCF method was used to detect levels of intracellular ROS compounds in myoblasts treated by oxidative stressor cumene hydroperoxide (CHP). CHP is one of the oxidizing agents used as an intracellular source of reactive oxygen intermediates [34]. As shown in Figure 7, DIP intercepts intracellular ROS compounds such as hydroxyl, superoxide or other peroxide-forming radicals, and eventually prevents the DCF fluorescence intensity. CHP easily permeates through the cell membrane which, in the absence of DIP, causes DCF oxidation and its fluorescence intensity increment, whereas, DIP suppresses the ROS generation inside the cells.

#### Antitoxic potency of DIP

Antitoxic property of DIP, based on the protection of viable myoblasts against cytotoxic and lethal effects of CHP, was evaluated using MTT assay. CHP has been frequently used as a model compound to study both the organic hydroperoxides and the mechanisms driving oxidative cell injury in mammalian cells [21], [34]. For this purpose, cells were treated with different concentrations of DIP and then stimulated by CHP as a strong toxic organic hydroperoxide molecule. The highly ROS generating ability of CHP caused the compound to be vigorously toxic and lethal for myoblast cells (Figure 8). DIP helps to counteract the toxic effect of CHP.

### Computational IP data

Ionization potential (IP) correlates with the ability of the antioxidant to donate electrons. Low ionization potential (IP) values favorably raise the electron-transfer reactivity, while high IP values decrease the electron-transfer rate between antioxidant and free radicals. Table 1 refers that DIP has very low IP (∼132.0 kcal/mol) not only in gas phase but also in water solvent. Solvent effect has been estimated in the frame of Onsager reaction field model. The present results do support the more newly report on the excellent antioxidant activity of DIP compared to its 4 different derivatives based on the IP value of 132.16 kcal/mol [35]. It is interesting to note that the IP value of DIP is 20 kcal/mol, less than that of trolox. It means that DIP is much stronger electron-donor resulting its higher antioxidant activity compared to trolox and also confirms the experimental results of Figure 4.

## Discussion

During the oxidation process, the wide range of molecules, particularly lipids (RH), are easily oxidized to peroxyl radicals (ROO^•^). Free radical “chain reactions” involve three successive reactions as;

(2) RH→ R^•^ (initiation).

(3) R^•^+O_2_→ROO^•^ (O_2_ addition).

(4) ROO^•^+ RH→ROOH+ R^•^ (chain reaction).

Reaction 3 happens very fast while reaction 4 is much slower and forms a chain reaction. Antioxidants such as DIP are just able to block the slower reaction (reaction 4) and are known as radical “chain-breaking antioxidant”, accordingly;

(5) ROO^•^ + DIP →ROOH + DIP^•^ (trapping ROO^•^ radical).

The intermediate DIP^•^ can react with other stable substances such as RH or with reactive unstable free radicals such as ROO^•^, R^•^ and even itself (DIP^•^). If the DIP^•^ radical was stable and nonreactive, it would partly react with other free radicals such as peroxyl/alkyl/DIP^•^ in the case of encounter, but not with stable RH substrates;

(6) DIP^•^ **+** ROO^•^ [R^•^, DIP^•^] → ROO-DIP [R-DIP, DIP-DIP] (non radical yield).

Therefore, a good chain-breaking antioxidant must create long living nonreactive radical lacking invasive effects on other RH substrate. Producing and identifying DIP^•^ radicals by EPR and UV-Vis techniques (Figure 2&3) indicated its reasonable stability as to be an effective “chain-breaking antioxidant”. So, it may not react or may have slow reaction with substrates such as RH or ROOH. Therefore, the capability of cardiovascular drug DIP to produce long living DIP^•^ during free radical scavenging process suggests superb antioxidant activity for DIP with the least side effects towards biological substances such as cellular components.

However, the action mechanism of DIP to scavenge free radicals is still unclear. To consider the main mechanism for radical scavenging we have focused on the quantum mechanical calculations at the B3LYP theory level. The optimized 3D structural geometry of DIP is displayed in Figure 9. As expected from the crystal structure data [36], the pyrimido [5,4-d] pyrimidine system is planar (see Figure9). Moreover the piperidine nitrogens including N(41) and N(91) as well as the amine nitrogens N(ll) and N(61) with their substituents C(12), C(15) and C(62), C(65), respectively, are planer [36]. Our optimized structure confirmed this important planer property of DIP as is indicated by bond length and torsion angles around the N bonds (Table 2). Also the capability of forming an intramolecular hydrogen bond O(14)-H(14)... N(2) was compatible with crystal structure as well as with Borges et al. quantum mechanical findings [35] (see Table 2). These data indicate that our optimization is reliable to energy minimization for antioxidant parameters evaluation. It is believed that the phenolic aromatic antioxidants (ArOH) act primarily via hydrogen atom transfer (HAT) to reduce free radicals because of carrying a labile O-H groups [37];

(7) ROO^•^ + ArOH → ROOH + ArO^•^ (direct hydrogen-transfer).

Considering the chemical structure of DIP in Figure 1, the mechanism of HAT is not conceivable. DIP does not have any phenolic labile O-H moiety while it has four ethanolic O-H groups. Second remarkable antioxidant mechanism is proton-coupled electron-transfer (ET). In this way, DIP^+•^ is produced by electron transfer followed by deprotonation in solution to give the corresponding DIP^•^ radical. At first view, this mechanism is possible to occur from DIP because of having different N atoms, enabling deprotonation.

(8) ROO^•^+ DIP → ROO^−^ + DIP^ •+^ →ROOH + DIP^•^ (proton-coupled ET).

ET mechanism involves ionization potential (IP) value for chain-breaking antioxidant activity. Calculation of ΔIP value (IP_compound_ – IP_phenol_) confirmed that the electron transfer mechanism is responsible for radical scavenging activity of DIP. Wright et al. already showed below ΔIP = −36 kcal/mol, the mechanism is dominated by HAT, but for ΔIP above that point, reactions tend to be predominantly ET [37]. Table 1 shows that DIP significantly has larger ΔIP, which is more than 1.4 times above the point needed for possible appearance of ET mechanism. Thus, DIP has IP that brings certainly this molecule into the range where ET mechanism can become important. Also, Table 1 shows that DIP is significantly a better antioxidant, with ΔIP larger than trolox. Moreover to confirm these findings, additional calculations have been done. To analyze the possibility of HAT mechanism we have computed H atom abstraction from ethanolic OH groups. The optimized structures with relative DFT energies are displayed in Figure 10. Molecular energies suggest that the elimination of the ethanolic H atom from O-H will be a difficult process compared to electron and following H^+^ hydrogen donation. In fact, the bond dissociation energy for direct ethanolic H atom from O-H groups is higher than the electron transferring and following H^+^ hydrogen donation from DIP. Interestingly, electron-transfer process is easier with the lowest barrier energy. Therefore, direct hydrogen-transfer to reduce free radicals is not a favorable process, as this leads to the formation of DIP^•^ with high barrier energy. However electron-transfer process is favorable, as this leads to the formation of a very stable DIP^•+^ radical. Finally, this process facilitated proton donating via proton-coupled electron-transfer mechanism as indicated in Figure 10. These findings also support the ET mechanism that was discussed above based on ΔIP value. Furthermore, both the ferric ion reduction and electrochemical oxidation methods are general indicators for electron-transfer reactions that demonstrated ET feasible from DIP to reduce free radicals. These experimental findings in aqueous solution confirmed computationally-acquired lower IP value for DIP that indicates the high ET capacity of DIP. Hence, the proton-coupled ET mechanism should be mainly the radical scavenging mechanism of DIP drug that contributes to the “chain-breaking antioxidant” activity of DIP (Eq. 8). This process involves the production of long lived stable DIP^•^ radical. Also, the cell protection by DIP against peroxide-forming radicals and reactive ROS compounds, can be explained by easily production of stable DIP^•^ radical under the oxidant condition. Easy generation of stable DIP^•^ radicals demonstrates occurrence of two important phenomena in biological conditions driven by DIP drug;

First, DIP can easily suppress more active free radicals such as ROO^•^ at different oxidative condition of human body to generate stable DIP^•^ by proton-coupled ET reaction (Eq. 8). Second, because of the stability of DIP^•^ radicals, these compounds are nonreactive enough to react with biological molecules such as lipids or protein and nucleic acids. We believe that the generated intermediate DIP^•^ radicals are noninvasive for the cells. The MTT assays based on the antitoxic potency of DIP, confirmed this property of DIP in myoblast cells. It is possible to conclude that the ROS scavenging and antitoxic capabilities of DIP are progressed based on the proton-coupled electron-transfer mechanism. The mechanism is closely connected with production of nonreactive long lived DIP^•^ radicals that culminate in dose-dependency of the drug to protect myoblast cells.
